# Unlocking biochar impacts on abiotic stress dynamics: a systematic review of soil quality and crop improvement

**DOI:** 10.3389/fpls.2024.1479925

**Published:** 2025-01-13

**Authors:** Periyasamy Rathinapriya, Theivanayagam Maharajan, Ravi Jothi, Mayakrishnan Prabakaran, In-Bog Lee, Pyoung-Ho Yi, Seung Tak Jeong

**Affiliations:** ^1^ Horticultural and Herbal Crop Environment Division, Soil Management Laboratory, National Institute of Horticultural and Herbal Science, Rural Development Administration, Wanju-gun, Republic of Korea; ^2^ Division of Plant Molecular Biology and Biotechnology, Department of Biosciences, Rajagiri College of Social Sciences, Kochi, Kerala, India; ^3^ Microbial Safety Division, National Institute of Agricultural Sciences, Rural Development Administration, Wanju-gun, Republic of Korea; ^4^ Institute for Fiber Engineering and Science (IFES), Interdisciplinary Cluster for Cutting Edge Research (ICCER), National University Corporation Shinshu University, Ueda, Japan; ^5^ Department of Biomaterials, Saveetha Dental College and Hospitals, Saveetha Institute of Medical and Technical Sciences (SIMATS), Saveetha University, Chennai, India

**Keywords:** abiotic stress, biochar (BC), BC synthesis, crop improvement, soil properties, soil stress alleviation

## Abstract

Global agricultural challenges, especially soil degradation caused by abiotic stresses, significantly reduce crop productivity and require innovative solutions. Biochar (BC), a biodegradable product derived from agricultural and forestry residues, has been proven to significantly enhance soil quality. Although its benefits for improving soil properties are well-documented, the potential of BC to mitigate various abiotic stresses-such as drought, salinity, and heavy metal toxicity-and its effect on plant traits need further exploration. This review aims to elucidate BC production by highlighting primary feedstock’s and synthesis techniques, and examining its role in boosting soil decomposition efficiency and fertility, which are pivotal for sustainable crop growth. This review also discuss how BC can enhance the nutritional and chemical properties of soil under different abiotic stress conditions, emphasizing its capacity to foster crop growth and development in adverse environments. Furthermore, this article serves as a comprehensive resource for agricultural researchers in understanding the importance of BC in promoting sustainable agriculture, and addressing environmental challenges. Ultimately, this review highlights critical knowledge gaps and proposes future research avenues on the bio-protective properties of BC against various abiotic stresses, paving the way for the commercialization of BC applications on a large scale with cutting-edge technologies.

## Graphical Abstract

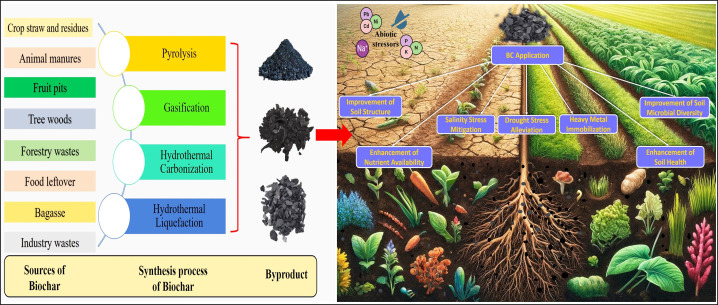

## Highlights

- BC ameliorates physico-chemical properties of soil against environmental stress.
- It serves as a significant nutritional reservoir for plant growth improvement.
- BC acts as a key player in mitigating abiotic stress tolerance for sustainable agriculture.
- Further studies are needed on expression pattern and characterization of genes in BC’s protective roles against abiotic stress.
- Emphasis should be placed on innovative uses of BC for agricultural stress management.

## 1 Introduction

Soil degradation, severe soil contamination and loss of soil fertility provoke a global threat to food security and agricultural sustainability. Rising food deficits and climate change need a green solution for improving soil quality and diminishing ecological agriculture impacts to ameliorate crop productivity. The excessive use of chemical fertilizers with salt and other acidic components reduces the productivity of crops by triggering soil quality via soil deterioration, soil acidity, and poor soil aggregate structures (Wu et al., 2023). Quality of soil is commonly affected by soil organic matter (OM), electrical conductivity (EC), and soil depth, which cause salinization, compaction, nutrient deficiency, erosion, loss of biodiversity and desertification, which all lead to soil fertility reduction (Dalal et al., 2011; Lal, 2015). In addition, soil nutrient depletion was directly associated with food insecurity due to unsustainable land use. To combat this, various soil additives are implemented to augment soil nutrients, including composts, inorganic chemical fertilizers, seaweed, organic manures, mulches, clay minerals, nanomaterials and sewage sludge, etc (Bibi et al., 2022; Yasmeen et al., 2022). Since most of these management approaches have less or no impact on the storage of soil carbon (C), prompt organic C (OC) decomposition results in the emission of carbon-di-oxide (CO_2_), thereby reducing the efficiency of C balance (Agegnehu et al., 2017; Chen et al., 2023). Hence, sustainable and reliable resource management techniques are urgently needed to mitigate global soil contamination and restore soil quality (Pradhan et al., 2018).

Global agricultural productivity has been significantly impacted by various abiotic stressors, which are major limiting factors affecting soil quality. Abiotic stressors such as drought, soil salinity, and heavy metal accumulation contribute to over 50% of crop production losses and affect 91% of the world’s cropland (Younis et al., 2020). Among these, heavy metal pollution, a notable consequence of anthropogenic activities, has significantly increased since the industrial revolution. For instance, high concentrations of heavy metals in soils adversely affect plant physiology, metabolism, and biochemical processes, leading to reduced growth, biomass, and yield of plants (Goyal et al., 2020). In addition to heavy metal pollution, reduced precipitation due to climate change has exacerbated global drought conditions. Drought stress reduces cell turgor and negatively impacts plant growth. It reduces shoot growth, limiting the production and transfer of photosynthetic materials, which ultimately decreases plant growth and yield (Nour et al., 2024). For example, severe drought stress reduced yield of rice (*Oryza sativa*) (53-92%), wheat (*Triticum aestivum*) (57%), maize (*Zea mays*) (63-87%), soybean (*Glycine max*) (46-71%) and chickpea (*Cicer arietinum*) (45-69%) (Fahad et al., 2017). Similarly, salinity stress reduces crop yield, with declines ranging from 5-50% due to osmotic stress, ionic toxicity, and nutrient imbalances (Okorogbona et al., 2015).

To meet the projected increase in food demand for an estimated global population of 9 to 10 billion people by 2050 (Van Dijk et al., 2021), various strategies have been implemented to improve crop performance under abiotic stress. These strategies include breeding techniques, agronomic practices, seed priming, microbial seed treatment, microorganism inoculation, grafting, and the use of plant growth regulators and osmoprotectants (Gupta and Shrestha, 2023; Oyebamiji et al., 2024). Especially, farmers use pesticides and hazardous chemical fertilizers to cultivate maximum crops in a minimal area, which further lowers land quality and causes soil degradation, contamination, erosion and water pollution (Cao et al., 2011; Oliver and Gregory, 2015; Pradhan et al., 2018). To combat this, the application of biochar (BC) has recently emerged as a cost-effective and environment friendly strategy to enhance crop tolerance to abiotic stress.

BC is a highly stable carbonaceous residue resulting from the thermochemical degradation of various feedstocks, such as crop residues, mill residues, agricultural wastes, food wastes, animal manure, and forestry wastes (Tomczyk et al., 2020). BC is valued for its micro-pores and high cation exchange capacity (CEC), which do not exacerbate environmental conditions. It is primarily composed of oxygen (O_2_), nitrogen (N), hydrogen (H), C, and aromatic and alkyl matter (Nath et al., 2022). BC’s unique structural and functional properties make it a valuable soil amendment for enhancing soil fertility. In USA and China alone, approximately 1.4 BT of agro-biomass waste are generated annually, producing around 420 MT of BC per year (Godlewska et al., 2017; Nath et al., 2022; Kumar et al., 2023). BC has been shown to enhance crop growth and yield under abiotic stresses and in metal-polluted soils. Incorporating BC into infertile or nutrient-deficient soils can improve crop performance, benefit farmers, reduce the use of inorganic fertilizers, and support environmental conservation (Ding et al., 2016; Wani et al., 2022). Raw BC has a limited ability to absorb contaminants from highly polluted water (Jagadeesh and Sundaram, 2023). Furthermore, the small particle size of powdered BC makes it difficult to separate pollutants from the contaminated water (Sivaranjanee et al., 2024). Nowadays, several studies focus on synthesizing novel BCs using nanocomposites to remove aqueous contaminants in an effort to overcome these unfavorable factors (Li et al., 2023; Dong et al., 2023). This type of nanocomposite-based BC method helps to improve the physical and chemical properties of BC. For instance, the nanocomposite BC exhibits higher porosity, more surface active sites, increased stability, a larger specific surface area, and a wider range of applications compared to the unaltered BC (Pan et al., 2021). Various review articles have well described the synthesis of BC nanocomposites (Chausali et al., 2021; Das and Panda, 2022; Lalhriatpuia and Tiwari, 2023). Hence, we can use this type of BC to remove heavy metal pollutants from wastewater and support efforts to improve the aquatic environment. To date, there is no much comprehensive review on the role of BC in alleviating abiotic stresses in plants. This review aims to highlight the production of BC from crop residual biomass and its wide range of applications under abiotic stress conditions. In addition, it provides updated information on BC for improving soil fertility and crop growth. Furthermore, we have discussed what studies should be carried out to understand the exact role of BC in crop development against abiotic stresses. Overall, we believe this review enhances the understanding of the role of crop residue-derived BC in controlling hazardous chemical-based soil amendments, reducing anthropogenic gas emissions, and ensuring environmental sustainability. Apart from this, this review will raise awareness in plant molecular biology researchers to initiate in depth molecular experiments to study about gene regulations on using BC for plant growth.

## 2 Methodology

Published research articles related to the topic were collected from five scientific databases, including Web of Science, “Scopus,” “PubMed,” “Science Direct,” and “Google Scholar”. The following combinations of search terms were used to collect articles: “role of BC on improving soil properties,” “biochar role in physical and chemical properties of soil,” “BC synthesis methods,” “Effects of biochar on drought stress tolerance in plants,” “Effects of biochar on salinity stress tolerance in plants,” “Effects of biochar on heavy metal stress tolerance in plants,” and “Role of biochar for improving abiotic stress tolerance in plants.” This review included articles published in English up until August 2024. Article titles and abstracts were manually assessed to exclude reports that were not relevant to this review. This review includes only biochar-related topics that enhance abiotic stress tolerance. We excluded only articles published in languages other than English.

## 3 Feedstock for BC synthesis

Effective selection of feedstock biomass is crucial for optimizing the preparation and yield of BC. Biomass feedstock, a complex solid material, can be categorized as either woody or non-woody. The classification of biomass applied for BC production is mainly based on its source, biological diversity and origin. Plant biomass and organic waste are the two main sources of feedstock used for BC production. In organic waste feedstocks, the use of various types of crop residue biomass for BC production has gained attention due to its economic benefits, environmental advantages, and scientific interest (Awogbemi and Kallon, 2023). Crop residue biomass includes materials that are not classified as processed or field residues, such as leaves, straw, stalks, shells, molasses, roots, husks, peels, bagasse, tree prunes, and pods, sourced from agricultural lands, homes, and industries.

Straws, for instance, can be converted into bioplastics, chemicals, biogases, enzymes, and biocatalysts (Bilo et al., 2018). Global estimates indicate that rice straw production is around 800-1000 MT, while wheat straw production is approximately 354 MT, consisting of cellulose (32-47%), lignin (5-24%), and hemicellulose (19-27%) (Bilo et al., 2018; Ingrao et al., 2021).

Bagasse, a multicellular lignocellulosic residual fiber extracted from sugarcane and other sources such as pomegranate, pineapple, cashew, and sorghum, is another significant biomass. In 2021, the production of sugarcane reached 1.6 BT, generating 279 MMT of sugarcane bagasse. Bagasse has numerous applications, including biofuel, ceramics, cement additives, bricks, catalysts, concrete, adsorbents, food additives, silage feed, and organic manure (Awogbemi and Kallon, 2023).

Pruned branches from fruit trees like apples, pears, and plums are abundant sources of lignocellulosic biomass. Farmers often incinerate these branches to reduce insect pests and plant diseases, contributing to atmospheric CO_2_ emissions (Sasaki et al., 2014). In 2010-2011, 1650 tons of pruned pear branches were reported to be discarded and accumulated in fields by the Tokushima Agriculture, Forestry, and Fisheries Technology Support Center of the Fruit Tree Research Institute (Sasaki et al., 2014). Utilizing pruned branches holds potential for BC and biofuel production.

The characteristics of BC derived from diverse crop residues have been compared in numerous studies (Wu et al., 2012; Windeatt et al., 2014; Purakayastha et al., 2015). For instance, Purakayastha et al. (2015) prepared four types of BC from maize stover, pearl millet stalk, rice straw, and wheat straw, finding that maize BC had higher nutrient values, particularly N and phosphorus (P), and greater C stability compared to other crop-derived BC. The study also determined that the total C content was highest in maize BC (66%), followed by pearl millet BC (64%), wheat BC (64%), and rice BC (60%). Another study utilized eight different crop-derived feedstocks, including coconut husk, coconut shell, cotton stalk, olive pomace, palm shell, rice husk, sugarcane bagasse, and wheat straw, for BC production, yielding 28% to 39% of BC. This study also found that high lignin feedstocks produced high-C BC with significant recalcitrance (Windeatt et al., 2014). Wu et al. (2012) demonstrated that rice straw-derived BC had high alkalinity, CEC, and levels of available P and extractable cations, indicating its potential as a fertilizer and soil amendment. Comparative analysis of BC production from various crop residue feedstocks is still limited, and it is necessary to identify the most suitable feedstocks for high-value BC production on a large scale.

**Figure 1:**
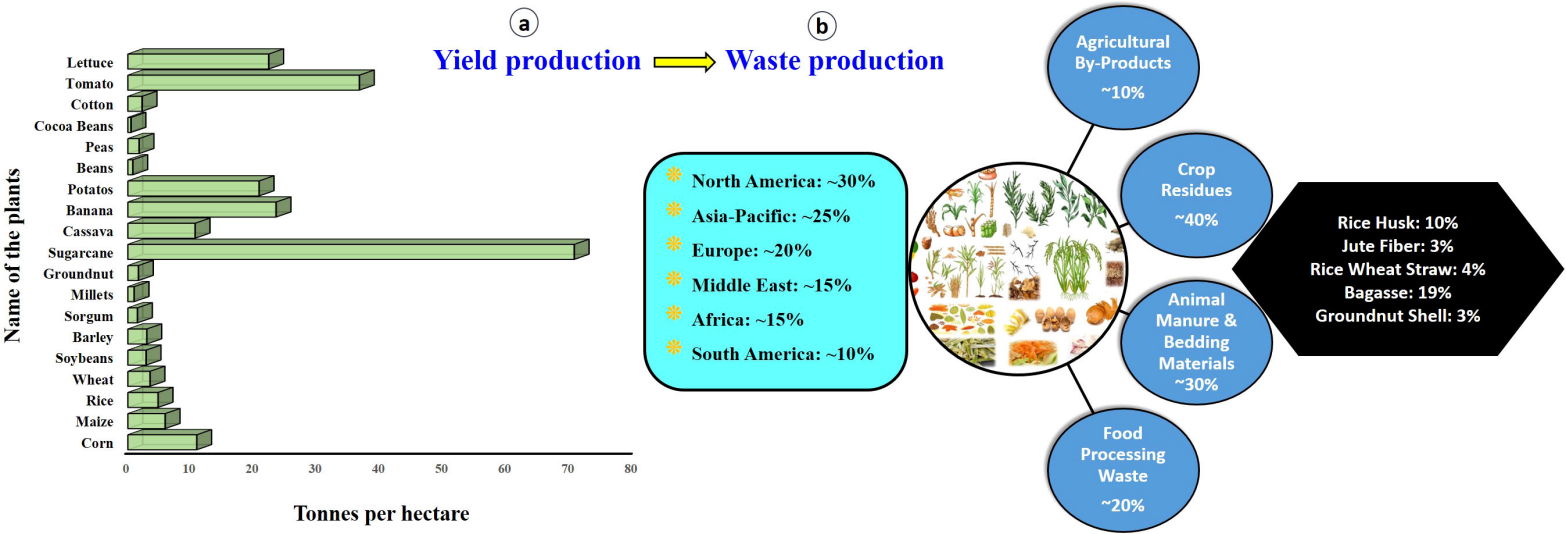
Data of annual food crop yields **(A)** and major wastage production **(B)** (FAO, 2022; MMR, 2023).

While specific data on the annual production of crop residues are unavailable, recent statistics (FAO, 2022; MMR, 2023) indicate that the global cultivation of primary crops increased by 52%, fruits by 55%, and vegetables by 65% between 2000 and 2020 (**Figure 1**). This significant increase suggests a corresponding rise in crop residue production each year. Converting these residues into BC is a vital sustainable waste management strategy that mitigates climate change, enhances plant growth, and protects the environment.

## 4 BC synthesis methods

BC can be synthesized through various thermochemical techniques, mainly pyrolysis, gasification, hydrothermal carbonization (HTC), and hydrothermal liquefaction (HTL). The production conditions and physico-chemical properties of used biomass resources for BC synthesis play a vital role in porosity, CEC, specific surface area, functional groups and yield percentage (Nath et al., 2022; Singh et al., 2022). **Table 1** presents the different techniques used for BC synthesis.

**Table 1:** Details of BC production techniques and advantages.

Synthesis technique		Description	Advantages	Reference
Traditional approach		Firebrick pits, clay heater, iron and brick retort furnace are used as a reactors and feedstock burnt directly in an open field covered partially with half burned biomass or soil to reduce oxygen supply	Low cost No energy consumed Advanced technical skills are not required	Thines et al., 2017; Gabhane et al., 2020; Masek, 2022
Conventional pyrolysis	Slow	TR: 300-600 °C; HR: 5-7 °C; min^-1^ RT: 60-120 min O_2_ supply: Nil	Energy consumption was less Moderate TR	Mendez et al., 2015; Awogbemi and Kallon, 2023
	Fast	TR: >500 °C; HR: 300 °C; min^-1^ RT: 0-20 min O_2_ supply: Nil	Conversion rate higher Low RT Better yield Higher amount of bio-oil produced Large-scale BC synthesis	Mendez et al., 2015; Laird et al., 2017
	Flash	TR: >1000 °C; HR: 1000 °C; sec^-1^ RT: 0-1 min O_2_ supply: Nil	Fast, effective, and efficient technique	Gabhane et al., 2020; Adelawon et al., 2022
Gasification		TR: >700 °C; Gasifying agents: Air, steam, O_2_, and CO_2_	Humid biomass was used Produced high quality BC, C_2_H_4_, C_2_H_2_, other useful fuels and eco-friendly chemicals Efficacious environmental, and economic benefits	You et al., 2018; Dafiqurrohman et al., 2022; Zhang et al., 2022
HTC		TR: 150-350 °C; Pressure: 2-10 MPa RT: Several hours O_2_ supply: Less or Nil	Biomass predrying not required Wet biomass can be directly converted into byproducts Enhanced hydrophobicity or dewater ability of feedstock	Pauline and Joseph, 2020; Chi et al., 2021; Moreira et al., 2021
HTL		TR: 250-374 °C; Pressure: 2-25 MPa	No predrying of feedstock required Recovers <70% as bio-oil and BC Less water usage	Gollakota et al., 2018; Chi et al., 2021

TR, temperature range; HR, heating rate; RT, residence time; HTC, hydrothermal carbonization; HTL, hydrothermal liquefaction.

### 4.1 Pyrolysis process

Pyrolysis is an ancient method frequently used for synthesizing BC from biomass in chemical or thermal conversion methods. Pyrolysis is the thermal decomposition of biomass under elevated thermal vibration. During pyrolysis or incineration with a low O_2_ supply, crop residue breaks down organic components into condensable liquids, noncondensable gases, and char (Jung et al., 2019). Generally, pyrolysis products are composed of flammable methane (CH_4_), ethane (C_2_H_6_), H_2_, and (carbon monoxide) CO syngas, and liquid products are composed of phenolics, furanics, fatty acids, fine chemicals, biofuels, and solid material of BC (Fahmy et al., 2020; Wang et al., 2020a; Cho et al., 2023). In accordance with the TR, HR and, RT, conventional pyrolysis can be classified as slow, fast, ultrafast, or flash pyrolysis. In slow pyrolysis, the ranges of HR 5-7 °C; min^-1^, RT 60-120 min and TR 300-600 °C; can predominantly yield 20-30% syngas, 25-35% bio-oil and 35-45% BC, respectively. Fast pyrolysis occurs without O_2_ at HR 300 °C; min^-1^, RT 0-20 min, TR >500 °C; and synthesis 20% of syngas, 60% of bio-oil and 20% of BC. Ultrafast or flash pyrolysis carried out in a fluidized bed reactor with HR ≥1000°C sec^-1^, RT ≤1 min, TR ≥1000°C yields solid 10-15%, liquid 70-80%, and gas 5-20%. This ultrafast pyrolysis limits wide industrial application since it produces high bio-oil but low levels of BC (Laird et al., 2017; Adelawon et al., 2022; Awogbemi and Kallon, 2023).

The BC yield during the pyrolysis process depends on the type and nature of biomass used. Temperature is the main operating process condition that decides the product efficiency. Generally, the yield of BC decreases and the production of syngas increases when the temperature is increased during the pyrolysis process. For instance, study illustrated that the molecular properties and Cu sorption capacity of BC, derived from Jerusalem artichoke stalks, are closely related to the temperature of pyrolysis (Wei et al., 2019). In that study, the content of O_2_-containing functional groups in the BC samples decreased, while that of aromatic structures and alkaline mineral components increased, with a rise in pyrolysis temperature (Wei et al., 2019). However, Sawargaonkar et al. (2024) confirmed that peanut shell BC obtained through slow pyrolysis process has greater BC yield as compared to the fast pyrolysis, irrespective of reaction temperature, thus it confirms the effectiveness of the slow pyrolysis mechanism toward the BC production. Wan et al. (2014) compared the characterization of BC derived from rice husk and elm sawdust by fast pyrolysis. They demonstrated that high in ash, while low in volatile and fixed C content found in rice husk derived BC compared to elm sawdust derived BC. This study represents the characteristics of BC was mostly determined by sources of feedstock rather than synthesis process. In general, BC sizes were varied in the range <150 to 2000 µm in pyrolysis process (Liu et al., 2017a; De Jesus Duarte et al., 2019).

### 4.2 Gasification

Gasification is an effective waste management process comprising steps like drying, pyrolysis, combustion, and partial oxidation (You et al., 2018; Siwal et al., 2020; Ajorloo et al., 2022). During gasification, partial oxidation enriches the chemical and textural properties of BC (Yaashikaa et al., 2020). At temperatures ranging from 700-1500°C, the heating rate is rapid, and the reaction duration varies from seconds to minutes, yielding ˜ 85% gas, 10% liquid, and 5% solid (Ambaye et al., 2021). Depending on availability, air, steam, CO_2_, O_2_, and their mixtures used in gasification, significantly enhance BC’s physico-chemical properties, biomass conversion efficiency, product composition, and gas synthesis (You et al., 2018; Maya et al., 2021).

Generally, the BC yield from gasification is lower than that from pyrolysis, this was attributed to C conversion to CO under partial oxidation conditions. Additionally, gasification BC has smaller specific surface areas and total pore volumes compared to slow and fast pyrolysis BC, mainly due to ash melting (pore clogging), pore expansion and collapse, and tar deposition at high combustion and reduction temperatures. However, previous study indicated that higher temperatures and varied gaseous conditions in gasification led to lower BC yields but larger total surface area, higher pH and ash contents, and very low tar content (16-polycyclic aromatic hydrocarbons) (Fryda and Visser, 2015). The particle sizes of gasification BC ranged from under 45 μm to over 2000 μm, showing inconsistency across studies (Griffith et al., 2013; Pujol Pereira et al., 2016; Shen et al., 2016).

### 4.3 Hydrothermal carbonization

HTC is a highly effective method for converting wet biomass into valuable byproducts without the need for pre-drying. The process operates within a reactor under pressures of 2-10 megapascal (MPa) and temperatures ranging from 150-350 °C;, with minimal or no O_2_ present, and lasts for several hours. The HTC process involves hydrolysis, dehydration, decarboxylation, aromatization, and re-condensation, producing syngas, hydro-char, and bio-oil (Chi et al., 2021; Awogbemi and Kallon, 2023).

Similar to pyrolysis, HTC generates a solid product of BC (called hydro-char), which makes up to 50-80%, along with a bio-oil and water mixture (5-20%), and CO_2_ (2-5%) (Saqib et al., 2019). However, hydro-char produced via HTC typically not classified as BC due to insufficient reaction temperatures, low C content, and an unfavorable O/C and H/C ratio (Wiedner et al., 2013). Recent research shows that combining HTC with pyrolysis can enhance BC quality and stabilize heavy metals in the final solid product (Li et al., 2022a). Olszewski et al. (2019) found that pre-treating brewery spent grains with HTC before pyrolysis significantly improves BC yield and C content, while reducing ash composition. Garlapalli et al. (2016) also observed an increase in C content to 82% in BC produced from the combined HTC and pyrolysis process, compared to 70% from HTC alone. Overall, improving hydro-char is crucial due to its low surface area (<30 m²/g), poor porosity, and the presence of harmful chemicals like furan, furfural, and phenolic compounds, which limit its use in soil improvement applications.

### 4.4 Hydrothermal liquefaction

In HTL, macro algae and lignocellulosic feedstock are broken down under high pressure and temperature in supercritical or critical water conditions to produce bio-oil, solids, gases, and organic byproducts. This process operates in a water medium at temperatures between 250-374 °C; and pressures of 5-20 MPa. HTL reactions involve the depolymerization of macromolecules, thermal decomposition, and recombination processes (Mathanker et al., 2021; Gollakota et al., 2018). The decomposition phase includes dehydration, decarboxylation, and deamination of the biomass, generating furfurals, phenols, soluble organic acids, polar organic molecules, and glycolaldehydes. Repolymerization and recombination are reverse processes of depolymerization, occurring upon the loss of H_2_ ions which act as free radicals. In the absence of H_2_, previously synthesized compounds repolymerize to form coke, a robust molecular complex (Ni et al., 2022; Ravichandran et al., 2022). HTL achieves a recovery of less than 70% of its feedstock as bio-oil and BC.

The high BC yield in HTL is estimated up to 70%, but have some challenges since biomass polymers are less likely to be converted to solid phases under hydrothermal conditions compared to other thermochemical processes like pyrolysis. Research indicates that the surface area and total volume of HTL BC are generally lower than those of pyrolysis BC, regardless of the feedstock used (Guo and Rockstraw, 2007; Leng et al., 2015). The surface area ranges from 1.56 to 17 m²/g, the average pore diameter from 18 to 36 nm, and the total pore volume from 0.058 to 0.082 cm³/g (Kumar and Pant, 2015; Leng et al., 2015). Despite these differences, HTL BC retains functional groups and volatile organic matter crucial for the adsorption of metals, dyes, and other pollutants. While HTL demonstrates efficient performance, economic viability, and a high production rate, it still faces numerous operational and technical challenges that hinder its full commercialization.

**Figure 2:**
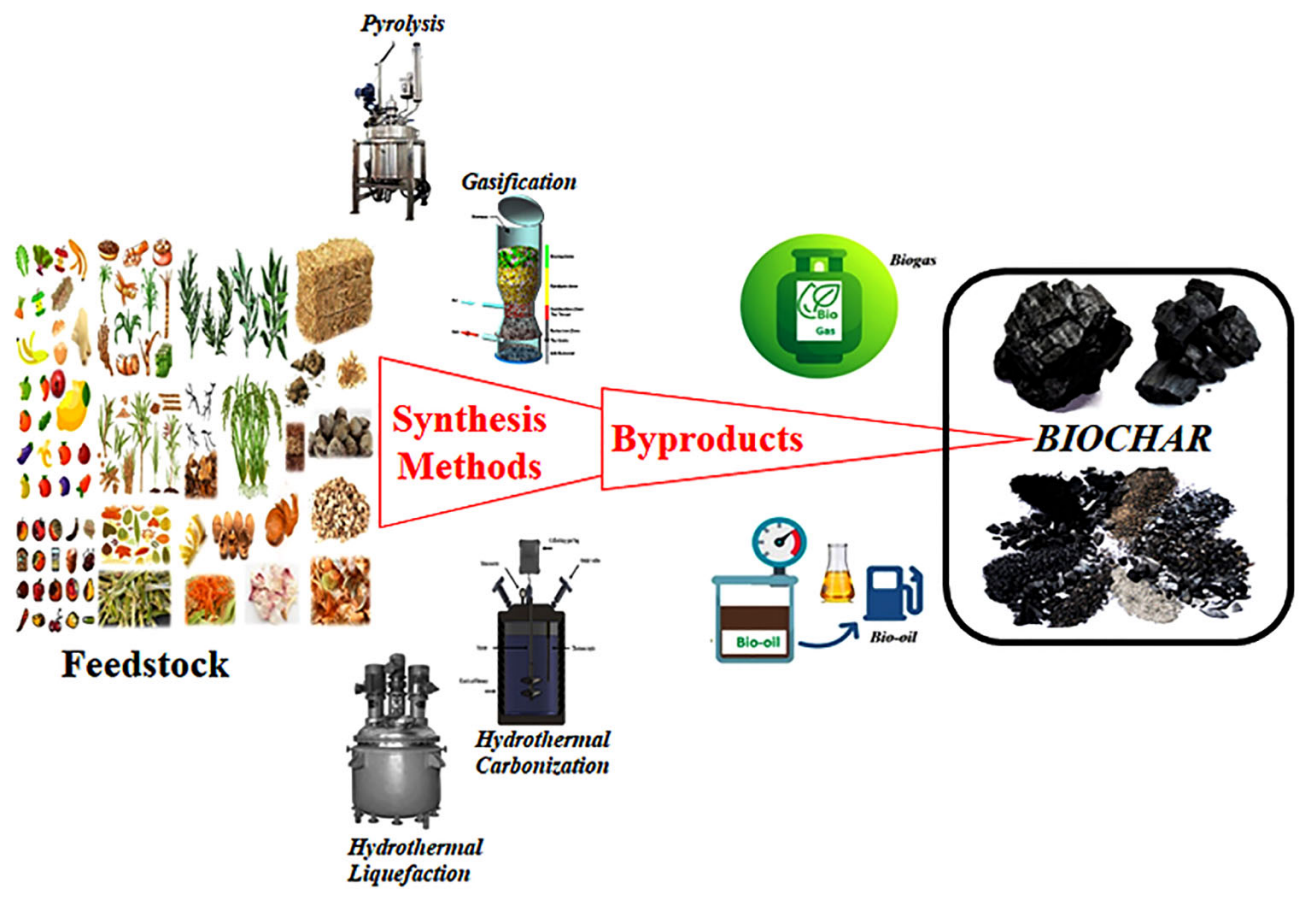
Schematic representation of various BC synthesis methods from different agricultural biomass residues. Feedstock is one of the major components for byproducts of BC. Four common thermochemical methods (Pyrolysis, gasification, hydrothermal carbonization and hydrothermal liquefaction) are widely used for synthesis of BC. Of these, pyrolysis is the most commonly used to produce BC.

Most of the above-discussed biomass-derived BC synthesis processes are less expensive, more convenient and farmer-friendly approaches than typical activation methods. A schematic representation of various BC synthesis methods can be seen in **Figure 2**.

## 5 Influence of BC physico-chemical soil properties

Improving soil health and adopting sustainable practices will boost crop yields, ensuring food security and environmental sustainability for the future. The impact of BC on soil properties has been widely studied. Morphological characteristics (e.g., large surface area and highly porous structure) of BC can change soil physical and chemical properties, which have been linked to changes in soil microbial community.

### 5.1 Impact of BC on physical properties of soil

Using BC as a soil amendment significantly increased various physical properties of soil such as BD, TP and WA.

#### 5.1.1 Soil bulk density and total porosity

The application of BC has been shown to significantly reduce BD and increase TP by indirectly influencing soil aggregation (Li et al., 2024). This process begins with a reduction in BD, followed by enhanced soil aggregation, interaction with mineral soil particles, and ultimately decreased soil packing. Lower BD can enhance soil structure, improve nutrient release and retention, and reduce soil compaction. Similarly, higher TP provides essential space and oxygen for soil organisms, influencing the transformation, storage, and utilization of water.

Studies have indicated that BC amendment improves BD, agglomerate stability, and aggregate capacity, thereby enhancing water retention and preventing soil degradation (Wang et al., 2020b). Consistent with Zhang et al. (2012a, 2012b), the addition of 40 t ha^-1^ rice straw BC to the soil reduced BD from 0.1 to 0.06 g cm^-3^ in 2009 and 2010, while increasing rice yield by 9-12% and 9-28%, respectively. Changes in TP were observed in the 5–10 and 25 μM ranges following BC addition (Rasa et al., 2018). Furthermore, BC implementation significantly enhanced soil permeability and saturated hydraulic conductivity (Oguntunde et al., 2008).

Jeffery et al. (2011) found that BC supplements significantly enhanced crop productivity in soils with acidic pH (14%), neutral pH (13%), and coarse (10%) or medium textures (13%). Sun and Lu (2014) found that straw bulk BC significantly increased pore volume in the macropore (> 75 μm) and mesopore (30-75 μm) ranges, likely due to the reorganization of pore-size distribution and aggregation processes induced by BC addition.

Overall, the impact of BC on BD and TP is closely related to the type of BC, soil type, BC particle size, and application rate. For example, Verheijen et al. (2019) demonstrated that smaller BC particles more effectively reduced the BD of sandy soil, while larger BC particles had a greater effect on reducing the BD of sandy loam soil, indicating that various BC particle sizes can be used to achieve specific soil effects. Similarly Rasa et al. (2018) highlighted that BC chemistry and pore morphology influence BC-water interactions, thereby altering soil textures accordingly.

#### 5.1.2 Water availability

Studies have demonstrated that BC significantly enhances WA in both sandy and clay soils (Ma et al., 2016; Pu et al., 2019). This is attributed to the porous nature of BC, which allows it to absorb substantial amounts of water, thereby altering the overall soil structure. In sandy soils, incorporating BC particles of various sizes and shapes can reduce the large gaps between soil particles (interpore spaces) and increase the proportion of micropores (5 to 30 µM in diameter) formed by the intrapores of BC. Consequently, when BC-sand mixtures become moist, the elongated shape of BC particles disrupts the grain packing in the sandy matrix, enhancing the interpore volume available for water storage (Liu et al., 2017b; Lehmann and Joseph, 2024). Applying BC at rates exceeding 3% *w/w* has been shown to potentially increase WA in clay soils (P < 0.05) (Kameyama et al., 2016). In a meta-analysis by Razzaghi et al. (2020) reported that BC additions increased available water content by 45% in coarse-, 21% in medium- and 14% in fine-textured soils. In clay soils, BC additions generally improve hydraulic conductivity and field capacity while reducing BD, thereby enhancing drainage, porosity, and plant-available water.

### 5.2 Impact of BC on chemical properties of soil

Soil chemical properties such as available N, P, potassium (K), pH, soil electrical conductivity was highly influenced by BC application.

#### 5.2.1 Nutrient availability

The soil environment is crucial for plant growth, and BC manifestation for long time showed to enhance soil nutrient availability and improve plants’ nutrient absorption efficiency. Therefore, BC can also be utilized as a vital nutrient source for plants and soil microorganisms. The higher levels of K, N, calcium (Ca), and P available in BC render nutrients to microorganisms essential for plant growth (Sakhiya et al., 2020). Hossain et al. (2020) reported that OC and essential minerals such as Ca, K, P, N, sulfur (S), and magnesium (Mg) were elevated via BC treatment in the soil. Gao et al. (2016) found that an increased retention of NO_3_ N (33%) and NH_4_^+^ N (53%) has a more significant effect in the soil upon BC amendment than direct nutrient supplements. Furthermore, BC application resulted in an increased grain P (38-230%) and N (20-53%) utilization efficiency compared to N fertilizer alone (Zhang et al., 2020a).

#### 5.2.2 Soil pH

BC application can also potentially modify common soil indicators such as pH, and electrical conductivity (Murtaza et al., 2023). Soil pH has a profound impact on plant growth and available nutrients. Generally, in agricultural fields, soil acidity (pH) increases through the application of lime to improve plant growth at maximum potential. The application of BC was found to rise the pH from 4.59 to 4.86 (Nielsen et al., 2018), from 4.8 to 6.3 (Novak et al., 2009), and from 4.3 to 4.6 (Hossain et al., 2010). Earlier studies showed that utilization of higher pH BC simultaneously increased pH in red ferralitic soil at approximately the 1/3 lime level, improved the Ca ratio and decreased Al toxicity (Glaser et al., 2002; Lehmann et al., 2003; Steiner et al., 2007). Granatstein et al. (2009) found that applying 39 t ha^−1^ herbaceous feedstock-derived BC to sandy soil increased the soil pH from 7.1-8.1. El-Naggar et al. (2018) found a significant increase in 71% electrical conductivity and a 5.2-7.6 pH range in sandy soils treated umbrella tree-derived BC compared to untreated controls. Mostly, BC was reported to not have any effect on light and highly acidic soils (Sousa and Figueiredo, 2016). Whereas, BC significantly increase the pH value of highly acidic to light alkaline soils (Boostani et al., 2020; Wen et al., 2022). Moreover, some studies revealed that increasing temperature during pyrolysis process has contributed to increase in soil pH by BC (Wan et al., 2014; Karimi et al., 2020).

#### 5.2.3 Cation exchange capacity and electrical conductivity

The CEC measures the soil’s ability to absorb, retain, and exchange cations. Enhancing the number of cation exchange sites in the soil can boost its CEC content. Soils with a high CEC are more capable of adsorbing NH_4_^+^, K^+^, Ca_2_^+^, and Mg_2_^+^, which enhances the efficient use of nutrient ions and minimizes nutrient loss (Liang et al., 2006). Higher CEC in soil supports plant nutrient cations binding to the clay, and humus to retain nutrients for uptake by plants instead of leaching (Glaser et al., 2002; Lehmann et al., 2003; Laird et al., 2010). After the addition of BC, the soil charge and CEC is reported to be increased by approximately 20–40% (Hossain et al., 2010). The anionic surface of BC was mainly attributed to increase the CEC of soil with both acidic and alkaline pH (Chintala et al., 2014). Tomczyk et al. (2020) found that woody BC enhanced the CEC of generated soil by 190% when compared with the untreated control. BC’s functional groups on the surface, silicon, alkalinity, and high pH-buffering capability contribute synergistically to moderate soil acidity (Mandal et al., 2020). The anion exchange capacity and CEC of the soil was also found to be emphasized by the incorporation of BC (Hossain et al., 2020).

### 5.3 Impact of BC on soil biological properties

The overall change of soil physical and chemical properties by the application of BC will result in the creation of appropriate habitat for living of beneficial microorganisms (Xu et al., 2014). In addition, due to the presence of high aromatic hydrocarbon and pore structure, BC served as a potential habitat and providing nutrient for various beneficial soil microorganism and resulted in improved crop productivity (Bolan et al., 2023). By increasing soil pH, BC renders the soil environment more beneficial for plant and microbes (Azadi and Raiesi, 2021). The micro and meso pores of BC stores water and dissolved substances required for the microbial metabolism. According to Li et al. (2022b), BC application has distinctive attributes, such as an altered strategy in root growth, enhanced enzyme activities and rhizosphere nutrient availability in soil. Plant growth regulators, karakins and other germination hormones released by BC trigger seed germination and soil physico-chemical properties (Kochanek et al., 2016). The efficacy of plant growth improvement with various BCs has been studied extensively (**Table 2**). The BC amendment augments soil microbial activity and diversity, which is fundamental for nutrient cycling, organic matter decomposition, and the overall health of the soil ecosystem (Hou et al., 2024). Microbial activity enhancement further aids in the stabilization of heavy metals and improves soil resilience to abiotic stresses (Pathy et al., 2020). Moreover, BC increases soil bacterial diversity and alter its structure (Huang et al., 2022).

**Table 2:** Ameliorative effects of various BCs on crop growth, development and yield.

Plant name	Botanical name	BC feedstock	Pyrolysis temperature	Level of BC	Effects of BC on plant growth enhancement	References
Pumpkin	*Cucurbita pepo*	Maize straw	350 °C	10 and 20 t ha^−1^	Improved leaf RWC	Langeroodi et al., 2019
Tomato	*Lycopersicon esculentum*	Cotton seed shell and rice husk	400°C	5% (*w/w*)	Increased RWC and leaf photosynthetic rate	Akhtar et al., 2014
Apple	*Malus domestica*	Rice husk	450 °C	80 g k^−1^	Increased seedling height, DW, respiration rate, higher root surface area, root length and root volume	Wang et al., 2019
Asian lotus	*Nelumbo nucifera* Gaertn.	Pinewood	–	10% (*w/w*)	Increased FW of leaf, root, DW of rhizome and relative Chl contents	Liu et al., 2016
Chickpea	*Cicer arietinum*	Red sage	450°C	3.5 t ha^-1^	Higher seed yield, haulm yield, and biological yield	Meena et al., 2023
Sweet basil	*Ocimum basilicum*	Black cherry wood	450°C	2 and 3% (*w/w*)	Seed germination increased, improved Chl contents, enhanced surface area, total root volume and length	Jabborova et al., 2021a
Ginger	*Zingiber officinale*	Black cherry wood	450°C	1, 2 and 3% (*w/w*)	Increased seed germination, leaf length, leaf number, DW of shoot and root	Jabborova et al., 2021b
Peanut	*Arachis hypogaea*	Maize straw	600°C	10 and 20 t ha^−1^	Photosynthesis, Chl fluorescence, and yield	Wang et al., 2021
Tomato, Radish, Lettuce and Sweet pepper	*Raphanus sativus, Lactuca sativa* and *Capsicum annuum*	Maritime pine wood chips	600°C	2 kg/m2 = 10 t ha^−1^	Increased mean FW and improved fruit and vegetable yield	Gonzalez-Pernas et al., 2022
Maize	*Zea mays*	Hardwood	–	18.4 Mg ha^−1^	Highest grain yield and zero removal of residue	Rogovska et al., 2016
Tea	*Camellia sinensis*	Tea plants	550°C	20 g	Higher macronutrient contents such as N, P, and K, enhanced the leaf biomass, stem biomass, and stem diameter	Zou et al., 2023

RWC, relative water content; DW, dry weight; FW, fresh weight; Chl, chlorophyll.

### 5.4 Decomposition properties of soil

The chemical composition in feedstock obtained from different sources affects the biological decomposition of BC (Lehmann et al., 2011). The decomposition rate was significantly higher (mean: 0.025% day 1) in crop-derived BC than (mean: 0.007% day 1) grass-derived BC as a result of lower condensed and less C content. Furthermore, wood-derived BC (mean: 0.004% day 1) contains the lowest decomposition rate due to its high C content (Hilscher et al., 2009; Knicker, 2010; Singh and Cowie, 2014). Wang et al. (2016) found that BC decomposition rates increased logarithmically over time and were significantly influenced by soil texture, clay content, biomass feedstock, pyrolysis temperature, and process duration. Limited information is known about BC degradation, and the effects on the turnover of native soil organic matter, degradation duration and other cascading effects remain unclear (Lehmann et al., 2011; Ameloot et al., 2013; Lorenz and Lal, 2014). Previously, the application of BC in different soils has been reviewed; however, an updated overview of the persistence, degradation, and stability of BC-amended soil is still lacking. The main reasons attributed to the paucity of decomposition of BC in soil are insufficient insight to distinguish total soil CO_2_ efflux from other high CO_2_ efflux from dead plant residues, root-derived CO_2_, dissolved OC, soil OM, initial BC stock, and other soil pyrogenic C (Kuzyakov et al., 2009; Wang et al., 2016).

Overall, as a soil amendment, it enhances the biological and physico-chemical characteristics of the soil, especially over a long time, enriching soil aggregation, water holding capacity (WHC), pH, and microbial activity, which enhances overall soil quality. By enhancing the soil’s organic matter content, BC can support sustainable soil management and agricultural productivity. BC acts as a multifunctional soil amendment that not only enhances the soil nutritional profile but also provides a sustainable solution to mitigate the adverse effects of salinity, drought, and heavy metal stressors on agricultural lands.

## 6 Soil fertility and plant growth enhancement mediated by BC

The frequent application of chemical fertilizers can eventually affect soil fertility, which in turn additionally pollutes adjacent aquatic ecosystems (Jote, 2023). BC application has been an effective way to efficiently reduce the use of synthetic fertilizers, elevate N use efficiency, and promote sustainable agriculture (Gao and DeLuca, 2016). Furthermore, BC promotes plant growth along with higher biomolecule contents, which ensures healthy plantations with nutritionally enhanced crop yields (Tan, 2023). Previously, studies have shown that the nutrient availability to plants and their retention ability in the soil have been improved through enhancing soil CEC and surface oxidation characteristics, which low native organic matter improves soil C stability upon BC amendment (Karimi et al., 2020; Singh et al., 2022). Soil augmented with BC endured higher concentrations of P, N, K, Ca, Mg, and S than untreated soils (Jin et al., 2024; Adekiya et al., 2020). The addition of maize residue BC at a rate of 1-2% (*w/w*) enhanced total N, P, K, copper (Cu), iron (Fe), manganese (Mn) and zinc (Zn) (Choudhary et al., 2021). Similarly, the application of BC increased the total N, K and P in loamy and clay loamy soils (Nabavinia et al., 2015; Xiao et al., 2016). However, Yao et al. (2017) reported a conflicting result that the addition of maize stalk-derived BC (50 and 200 Mg ha^-1^) diminished total P and increased total N in soil. The combination and adequate concentration of soil mineral nutrients play a major role in the growth and development of plant species, and nutrient deficiency diminishes plant growth and yield (Alkharabsheh et al., 2021; Khan et al., 2024). All the reports revealed that the application of BC in soil increased the availability of both macro and micro nutrients.

Apart from improving soil quality, BC enhances seed germination and root development in various plants. For example, seed germination in sweet basil significantly increased by 28% in the 2% BC treatment and 30% in the 3% BC treatment compared with the non-BC control, which depicts a pivotal role of BC in seed germination (Jabborova et al., 2021a). However, improvement in seed germination depends on various factors, such as the type of BC feedstock, rate of BC application, plant species, soil, and other environmental conditions. BC incorporated in soil significantly improves root length, root size, surface area, root diameter, and root volume in apple, strawberry, and sweet basil compared with control plants (Wang et al., 2019; Chiomento et al., 2021; Jabborova et al., 2021a). Moreover, increasing (0, 5, 20, and 80 g kg^-1^) BC dosage application resulted in an increased root respiration of 745, 863, 960, and 1239 nmol O_2_ min^-1^ g^-1^ FW in apple seedlings, respectively (Wang et al., 2019).

BC application enhances plant growth regulators, seed germination, photosynthetic pigments, root growth and soil microbes, which are vital determinants of healthy plant development and productivity and synergistically improve the morphological, physiological, and biochemical properties of plants, soil enzymatic activities and soil fertility. Henceforth, BC may serve as a significant nutritional reservoir for crop development and an efficient amendment to improve soil characteristics.

## 7 Mechanisms of BC action in alleviating abiotic stresses in soil

Abiotic stresses significantly affect soil health and agricultural productivity. BC has emerged as a promising eco-friendly amendment for enhancing soil nutritional profiles under various abiotic stressors such as salinity, drought, and heavy metal contamination. Amendment of BC in saline soils enhances mineral nutrient, physical, chemical, and biological characteristics of the soil. BC boosts the availability of mineral nutrients and metabolism, EC, infiltration rate, BD, microbial biomass C and pH of the soil under saline-affected soils (Singh et al., 2022). BC ameliorates the adverse salinity effects by balancing WHC, porosity, and its high salt adsorption capabilities (De Vasconcelos, 2020). Specifically, in salt-stressed conditions, BC application was found to notably increase rice biomass through improving soil properties, nutrient conditions and reducing salinity indices like EC, soluble Na^+^, and Cl^-^ concentrations (Huang et al., 2022). Further, BC has shown to mitigate salinity and drought stress by improving soil structure, increasing water retention capacity, and enhancing the availability of water to plants. It alters the ionic balance in soil, reducing the uptake of Na^+^ ions under saline conditions and thereby promoting plant growth under stress (Zhang et al., 2013).

BC aids in preserving soil nutrients by diminishing nutrient leaching, in sandy and significantly weathered soils. The higher BC surface area and porosity serve as adhesion sites for nutrients. BC augments soil CEC, substitutes detrimental Na^+^ ions with beneficial K and Mg ions plays a crucial role in diminishing soil salinity (Bekchanova et al., 2024). Hence, it improves retaining of vital nutrients like K, Ca, and Mg availability to plants. The alkaline pH of BC plays a significant role in neutralizing acidic soils, thereby unravelling nutrients that are naturally inaccessible in acidic environments. The application of BC enhances the soil WHC, pH, CEC and decreases BD contributing to the reduction of heavy metals’ bioavailability and the alleviation of stress caused by salinity and drought (Kumari et al., 2020; Li et al., 2021a).

By increasing the soil pH, BC renders the soil environment more beneficial for plant and microbes. The adjustment of pH facilitates the solubility of nutrients that are less available under acidic conditions, such as P, and helps in reducing the toxicity of aluminium (Al), which causes problem in low pH soils (Huang et al., 2023). BC potentially mitigates heavy metals in saline soil through increasing soil organic C, microbial and biochemical activities (Azadi and Raiesi, 2021). The synergy between BC and soil organic matter is pivotal for stable soil aggregates formation that facilitates root penetration and improves water infiltration. Furthermore, BC porosity enriches soil porosity, BD, soil structure and water retention that alleviates plants’ resilience to drought conditions (Mukherjee and Lal, 2013).

Immobilization of heavy metals occurs through adsorption on BC surface, complexation with functional groups, and precipitation as metal-BC complexes, thereby mitigating the toxic effects of lead (Pb), cadmium (Cd), and chromium (Cr) in contaminated soils (Das et al., 2023). BC application is crucial for restoring the productivity of soils affected by industrial pollution and mining activities. BC significantly diminishes metal uptake by plants, as evidenced by lower concentrations of metals in plant tissues.

**Figure 3:**
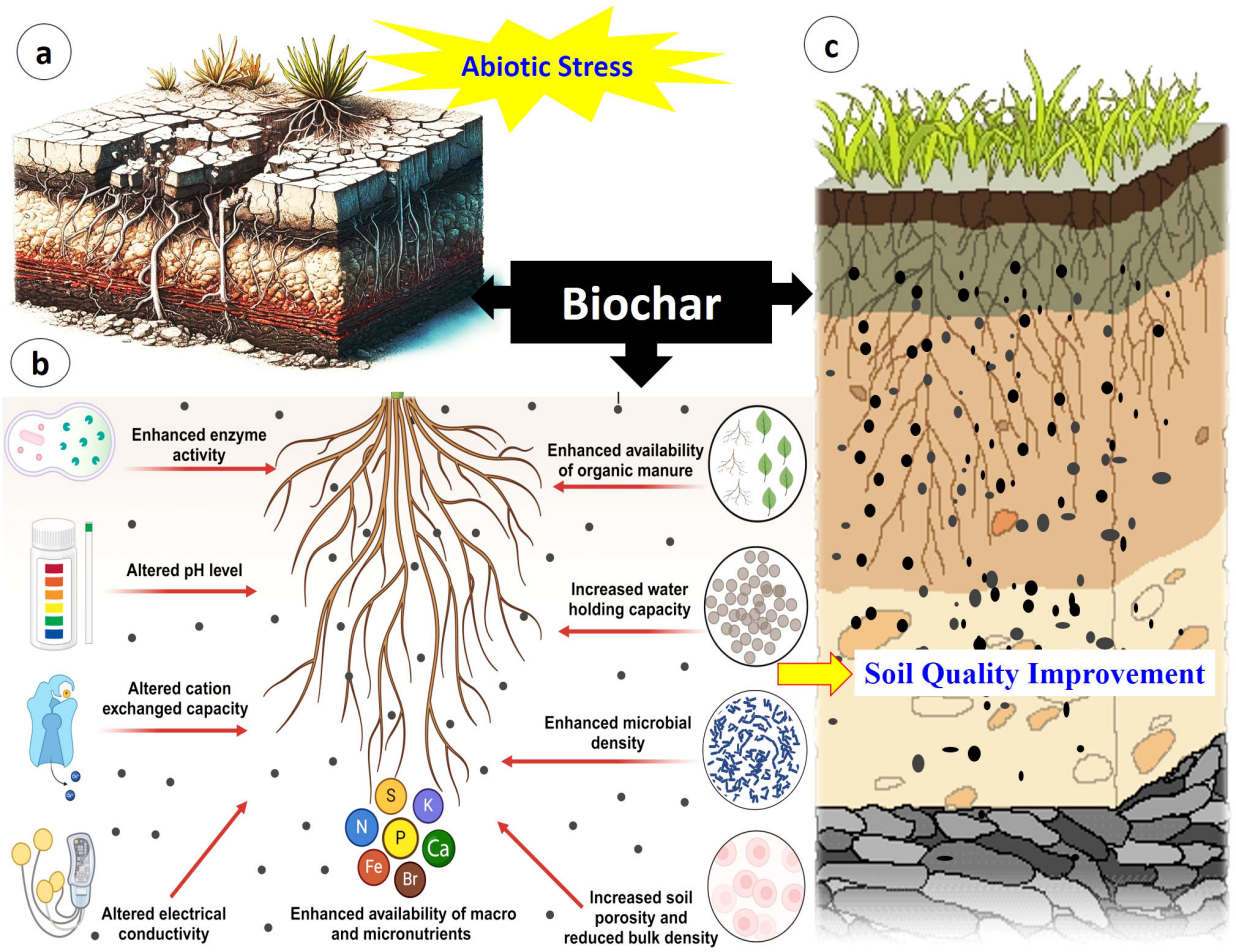
An illustrative effect of soil under abiotic stresses and BC amendment on soil characteristics. **(A)** Soil quality is severely affected by several abiotic stresses such as drought, salinity, heavy metals and nutrients deficiency, which directly reduce the growth and yield of any plants. **(B)** Application of BC to soil improves soil quality through various processes. For example, BC enhances availability of organic manure, macro and micro nutrients, microbial density and water holding capacity. In addition, BC maintains soil pH levels and modifies CEC and electrical conductivity. All these changes favor the conversion of infertile soil to fertile soil **(C)**, which helps improve plant growth and yield under severe abiotic stresses.

The ability of BC to enrich nutrient retention in soil is largely attributed to its unique surface functional groups and porosity. The carboxyl, hydroxyl, and phenolic functional groups on BC’s surface are pivotal for enhancing the soil’s ability to retain nutrients, particularly under nutrient-stressed conditions (Hagemann et al., 2017; Pandit et al., 2018). BC functional groups with its porous structure significantly regulates soil P, and N retention by influencing microbial dynamics, which is vital for plant growth (Ibrahim et al., 2020; Zhang et al., 2021). BC can protect organic matter from decomposition, leading to increased soil C sequestration. This stabilization of OM contributes to the long-term improvement of soil fertility, structure, and nutrient cycling (**Figure 3**).

## 8 Role of BC in mitigating various abiotic stresses for plant growth and development

**Figure 4:**
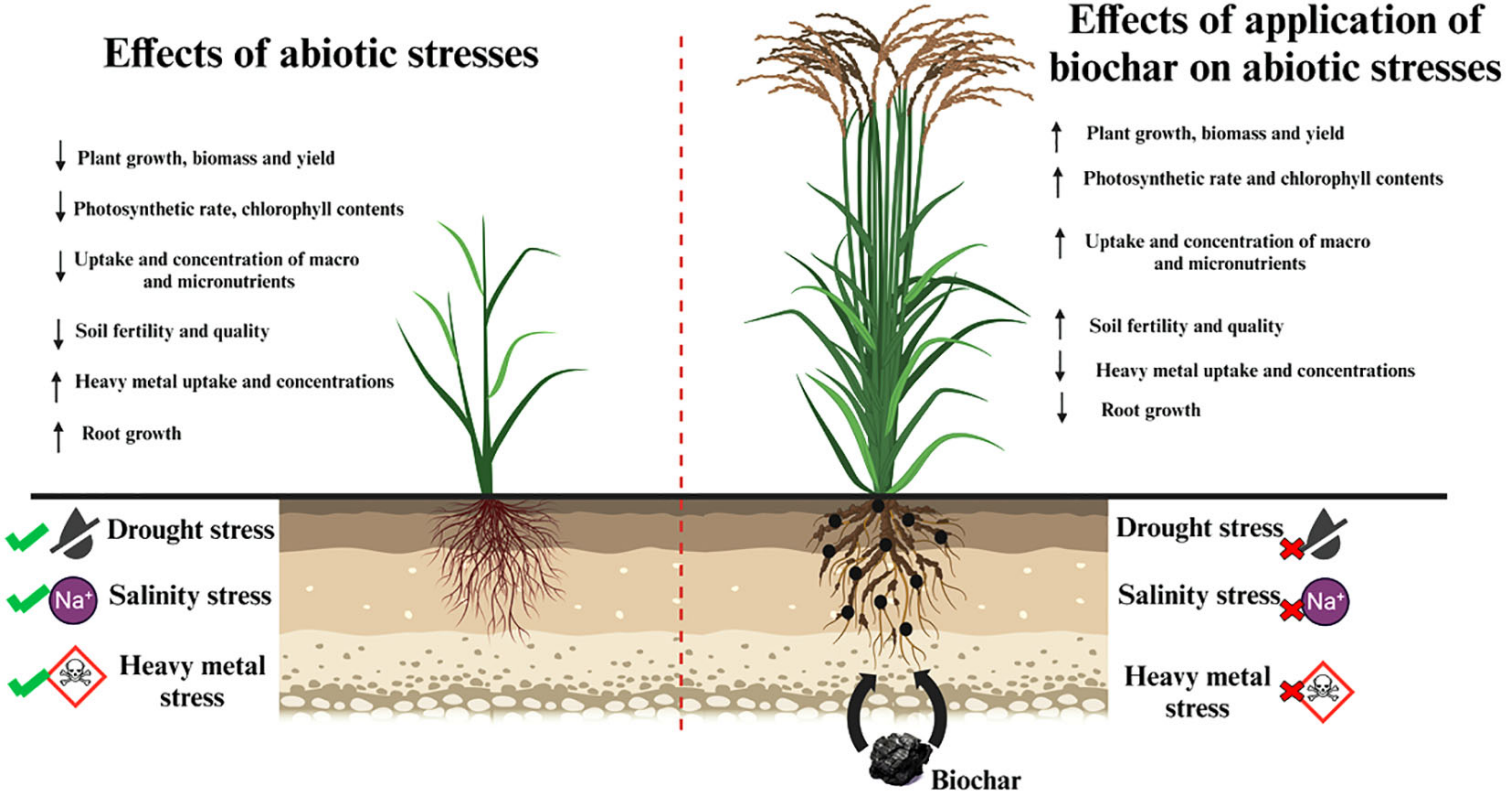
A visual demonstration of the positive impact of BC application on plants grown under normal and different abiotic stresses.

Abiotic stresses have been the main constraints for crop production in recent years. For several decades, plant researchers have used various techniques to mitigate abiotic stresses for plant growth and development. In recent years, many researchers have suggested that the application of BC in soil helps to alleviate different abiotic stresses and supports the enhancement of plant growth and yield. Therefore, BC is called “black gold” for agriculture. In this section, we discuss the role of BC in crop improvement under drought, salinity, and heavy metal conditions. **Figure 4** is a visual demonstration of the positive impact of BC application on plants grown under normal and different abiotic stresses.

### 8.1 Salinity stress

Higher concentrations of salt in soil induce osmotic stress due to ionic imbalance, which causes severe effects on morphology, biomass, yield, and biochemical processes in plants (Balasubramaniam et al., 2023). Salinity stress affects over 1000 million hectares of agricultural land worldwide, making it a serious threat to agriculture (Butcher et al., 2016). Therefore, eco-friendly technology is urgently needed to alleviate salinity stress in soil, which helps to improve crop growth and yield. BC enhanced plant biomass, root length, root volume, yield, leaf functional traits and K^+^ concentration in soybean, tomato (*Solanum lycopersicum*) and potato (*Solanum tuberosum*) under salinity stress (Akhtar et al., 2015; Farhangi-Abriz and Torabian, 2018a; She et al., 2018). Studies by Anwari et al. (2019a, 2023) demonstrated that BC treatment improved growth, biomass and yield traits of rice as well as soil properties (including nutrients availability) under saline conditions. Kanwal et al. (2018) found that applying BC increased the length of root and shoot, leaf functional traits and osmotic potential but decreased the proline content, superoxide dismutase activity and soluble sugar upon salinity stress. BC alleviated salt stress by maintaining higher leaf relative water content (RWC) and a lower Na^+^/K^+^ ratio and further enhanced the plant growth, biomass, photosynthesis, transpiration rate and grain quality of rice, sorghum (*Sorghum bicolor*), maize and wheat (Anwari et al., 2019b; Huang et al., 2019; Ibrahim et al., 2020, 2021). In another study, BC increased the plant stomatal conductance, plant yield, and chlorophyll fluorescence parameters and reduced abscisic acid in salinity stress-exposed cabbage (Chen et al., 2023). In addition, BC has high salt uptake ability, thus reducing Na^+^ uptake in plants and mitigating the adverse impact of soil salinity (Huang et al., 2019; Yang et al., 2020).

The role of BC in response to salinity stress in plant growth and metabolism has been extensively studied (**Table 3**). Overall, it can be concluded that BC can be a useful strategy to alleviate the harmful effects of salinity on plant development. However, BC rates must be carefully used in saline soil to reduce saline toxicity and enhance plant growth processes. The role of BC in physiological and biochemical responses under salt stress has not been studied in many horticultural and economically important plants. Hence, initiating further experiments using BC in other horticultural and economically important plants will help to improve plant quality upon salinity stress, which may help to reduce malnutrition worldwide. Moreover, numerous salinity stress-responsive genes are involved in improving plant growth under salt stress (Golldack et al., 2011; Kumar et al., 2017). The expression pattern and role of salinity stress-related genes have not yet been identified in plants grown under salinity stress with the application of BC. Henceforth, identifying the expression pattern of salt stress-responsive genes in various tissues of plants grown under salinity stress by the application of BC helps to determine which genes are highly induced by BC under salinity stress conditions.

**Table 3:** Effects of BC on plant growth and yield under salinity stress in various plants.

Plant name	Botanical name	Cultivars used	Concentration of NaCl	BC sources	Level of BC	Positive and negative effects of BC	References
Potato	*Solanum tuberosum*	Folva	25 and 50 mM	Commercial charcoal	5%	Increased root length, root volume, tuber yield, photosynthetic rate, intrinsic water use efficiency and K^+^ concentration Decreased leaf water potential, ABA content in xylem sap and leaf, Na^+^ concentration, leaf Chl content index, total leaf N and C content	Akhtar et al., 2015
Soybean	*Glycine max*	M7	5 and 10 dS m^−1^	Sycamore maple plant residues	50 and 100 g kg^−1^	Increased the nodule number and weight, DW of shoot and root, total plant biomass, total plant N, GDH, GS, GOGAT, and NO_3_^-^	Farhangi-Abriz and Torabian, 2017
Common bean	*Phaseolus vulgaris*	Derakhshan	6 and 12 dS m^−1^	Sycamore maple plant residues	10 and 20%	Increased DW of shoot and root, IAA content of roots Decreased ABA, ACC, JA contents and Na^+^ content of roots and leaves	Farhangi-Abriz and Torabian, 2018c
		Derakhshan	6 and 12 dS m^−1^	Sycamore maple plant residues	5 and 10%	Increased length of root, shoot and leaf area, DW of shoot and root, RWC, Chl fluorescence, Chl-a, Chl-b, total Chl and Chl a/b ratio and various ion concentrations (K^+^, Ca2^+^, and Mg^2+^) in root and shoot tissues Decreased Na^+^ concentration in shoot and root tissues	Farhangi-Abriz and Torabian, 2018b
Tomato	*Lycopersicon esculentum*	Yazhoufenwang	1 and 3 dS m^−1^	Wheat straw	2, 4 and 8%	Increased photosynthesis and transpiration rate, yield, and number of fruits	She et al., 2018
Wheat	*Triticum* *aestivum*	NARC 2009 and NARC 2011	150 mM	Wheat leaves	1 and 2%	Increased root and shoot length, leaf water potential and osmotic potential Decreased proline content, SOD activity and soluble sugar	Kanwal et al., 2018
		Sumai-10	0.3 and 10 dS m^−1^	Wheat straw	10, 20 and 30 t ha^−1^	Increased electrical conductivity, total above-ground biomass, grain yield, harvest index, spike and kernel number, 1000-grain weight, leaf RWC, photosynthesis rate and available P, N and K content in the soil Decreased Na^+^ concentration	Huang et al., 2019
Mung bean	*Vigna radiata*	MN92	5 and 10 dS m^-1^	Sycamore maple plant residues	50 and 100 g kg^-1^	Increased root length, root diameter, root DW, root density, specific root length, total root area, shoot DW, shoot/root ratio, root RWC, IAA in root Decreased ABA and ACC content in root	Nikpour et al., 2019
Sorghum	*Sorghum bicolor*	Kambal	0.26, 5.8, and 12.6 dS m^-1^	Wheat straw	2.5, 5, and 10%	Increased plant height, leaf area, FW, dry matter yield, photosynthetic rate, stomatal conductance, transpiration rate Decrease activity of CAT, POD, SOD	Ibrahim et al., 2020
		Kambal	0.8, 4.1 and 7.7 dS m^−1^	Wheat straw	2.5, 5, and 10%	Increased shoot and root length, FW and DW of shoot and root and RWC	Ibrahim et al., 2021
Eggplant	*Solanum melongena*	Jaylo	2 and 4 dS m^−1^	Oak and Pine tree woods	5%	Increased stomatal conductance, photosynthesis rate, density of root length and surface area, plant height, stem diameter, leaf area and yield Decreased leaf temperature and electrolyte leakage in the leaf	Parkash and Singh, 2020
		Bonica F1	300 mM	Maize straw	6%	Increased plant height, aerial biomass, fruit number per plant, flowering time and mean FW	Hannachi et al., 2023
Quinoa	*Chenopodium quinoa*	Titicaca	400 mM	Corn straw	5%	Increased plant height, shoot biomass, grain yield, leaf photosynthesis, stomatal conductance, intrinsic water use efficiency and leaf K^+^ Decreased total leaf water potential and ABA content in leaf, leaf Chl content index, leaf N and C content and leaf Na^+^ content	Yang et al., 2020
Rice	*Oryza sativa*	G9	352.11 mM	Wheat straw	15, 30 and 45 g per kilogram	Increased biomass, grain yield and quality Decreased Na^+^ ion accumulation of various tissues	Jin et al., 2018
		Changbai-9	23.91 dS m^−^	Rice husk	30g/kg	Improved rice grain quality traits including amylose content, protein content, taste value, rough rice grain, brown rice rate, white rice rate, whiteness and transmission rate and production	Anwari et al., 2019b
		Jinyuan 85 and Nipponbare	1 and 3 g kg^−1^	Rice straw	–	Decreased electrical conductivity, exchangeable Na^+^ and exchangeable Cl^-^	Zhang et al., 2019
		Changbai-9	368.11 mM	Peanut shell	33.75, 67.5 and 102.5 t ha^−1^	Increased leaf water status, plant height, chlorophyll content index and K^+^ concentration Decreased Na^+^ concentration, Na^+^/K^+^ ratio and leaf-relative electrical leakage	Ran et al., 2020
		Changbai-9	23.91 dS m^−^	Rice husk	–	Increased plant height, tiller number, dry weight of leaf, panicle, stem and sheath, total dry biomass, K^+^ concentration and K^+^/Na^+^ ratio Improved the concentrations of soil pH, Ca^2+^, Mg^2+^, CO32^-^ and Cl^-^ Decreased Na^+^ concentration in different rice organs and considerably	Anwari et al., 2023
		Tianlongyou 619	4.5 dS m^−1^	Maize, wheat and peanut shell residue	0.5 kg m−^2^	Increased plant height, DW, root length, and grain yield Reduced amylose, protein, and taste quality	Zhang et al., 2024
Maize	*Zea mays*	Xianyu335	2.0 and 5.0 dS m^−1^	Wheat residue	5 and 10%	Increased plant height, stem diameter, number of leaves per plant, photosynthetic rate, transpiration rate, yield and nutrients N, P, and K uptake	Alfadil et al., 2021
		Naudi hybrid	1.25 and 2.5 g L^− 1^	Eucalyptus residues	50 and 100 g kg^− 1^	Increased shoot and root length, DW of shoot and root, Chl a and Chl b, GSTs and CAT activities	Helaoui et al., 2023
Cabbage	*Brassica olerecae*	Yalova1	150 mM	Commercial charcoal	2.5 and 5%	Increased stem diameter, leaf area, FW and DW of root and shoot, leaf RWC, Chl a, Chl b, total Chl and plant nutrient uptake Reduced MDA, H_2_O_2_, proline, sucrose Na and Cl content	Ekinci et al., 2022
		Shanghai Green	25, 50, and 100 mM	Corn stover	2 and 4%	Increased the plant stomatal conductance, plant yield, Chl fluorescence parameters and reduced ABA	Chen et al., 2023

RWC, relative water content; DW, dry weight; FW, fresh weight; Chl, chlorophyll; ABA, abscisic acid; ACC,1-aminocyclopropane-1-carboxylic acid content; JA, jasmonic acid; IAA,indole-3-acetic acid; GDH, glutamate dehydrogenase; GS, glutamine synthetase; GSTs, glutathione-S-transferase; GOGAT, glutamine oxoglutarate aminotransferase; SOD, superoxide dismutase; CAT, catalase; POD, peroxidase; MDA, malondialdehyde.

### 8.2 Drought stress

One of the most important environmental factors affecting the entire plant life cycle was drought. Over 45% of the world’s cultivated land is permanently drought-prone, and 38% of the world’s population lives there (Adhikari et al., 2015). Therefore, improving water use efficiency in plants exposed to drought stress has long been an important factor in enhancing plant growth and development (Ruggiero et al., 2017). Soil application of BC is considered as an effective practice to facilitate plant growth and yield under drought stress. Many studies have shown that the application of BC in soil increases the growth and yield of drought-stressed plants (**Table 4**). In tomato, BC increased the soil moisture content, photosynthetic rate, yield, quality of fruit and other biochemical traits under drought stress (Akhtar et al., 2014; Obadi et al., 2023; Zhang et al., 2023).

**Table 4:** Effects of BC on plant growth and yield under drought stress in various plants.

Name of the plant	Botanical name	Cultivars used	Drought level	BC sources	Level of BC	Positive and negative effects	References
Tomato	*Lycopersicon esculentum*	No.2 Hongfen	28% WHC	Mixture of rice husk and shell of cotton seed	5%	Increased the soil moisture contents, photosynthetic rate, physiology, yield, quality of fruit, RWC, membrane stability index, water use efficiency, stomatal pore aperture and stomatal density Decreased leaf N content and Chl content index and ABA content in leaf	Akhtar et al., 2014
		Tone Guitar	40, 60 and 80% WHC	Date palm fronds waste	5%	Increased plant height, leaf area index, stem diameter, and FW and DW of above-ground tissues, Chl-a, Chl-b, total Chl, Car contents, water use efficiency and fruit yield	Obadi et al., 2023
		Ailsa Craig	70% FC	Wood and poultry manure BC	5%	Increased plant height, dry mass accumulation, dry root mass, dry leaf mass, ratio of root and shoot, specific leaf area, field WHC and soil water supply Decreased ABA content in xylem sap	Zhang et al., 2023
Lady’s Finger	*Abelmoschus* *esculentus*	–	60% FC	*Lantana camara* plant residues	1 and 3%	Increased photosynthesis, leaf area, stomatal conductance, dry matter, transpiration rate water use efficiency	Batool et al., 2015
Milk thistle	*Silybum marianum*	–	40% WHC	Sycamore maple hardwood	1 and 2%	Increased net photosynthesis rate, water use efficiency, membrane stability index, Chl-a, Chl-b, total Chl, leaf weight, stem weight, leaf area, plant weight and plant height Decreased internal CO_2_ and stomatal conductance	Afshar et al., 2016
Soybean	*Glycine max*	NARC II	–	Corn cobs	10 and 20 t ha^-1^	Increased seedling vigor, germination percentage, rate of germination, membrane stability index of leaf, RWC, shoot length, Chl-a and Chl-b, Car and total Chl Decreased sugar and proline content	Hafeez et al., 2017
		Zhonghuang 35	40–45% WHC	Wheat straw	5, and 10 g kg^− 1^	Increased water use efficiency, grain yield, root length, root and shoot biomass, photosynthetic rate, stomatal conductance and transpiration rate	Zhang et al., 2020b
		Yeşilsoy	50, 75 and 100 100% FC	Hazelnut shells	3 and 6%	Increased plant height, FW and DW of shoot and root, stem diameter, leaf area, Chl-a, Chl-b, total Chl, IAA and GA content Reduced MDA, H_2_O_2_, proline, ABA, and sucrose content, and antioxidant activities	Gullap et al., 2024
Maize	*Zea mays*	Amadeo and DKC-3399	25–30% WHC	Wood-chip sievings	1.5 and 3%	Increased above-ground biomass, water use efficiency and soil NO_3_^-^ content	Haider et al., 2015
		ICI-8914	40% WHC	–	4 t ha^−1^	Increased DW of shoot and root, length of shoot and root, net photosynthetic rate, transpiration rate, stomatal conductance, Chl-a, Chl-b and Chl a+b	Sattar et al., 2020
Wheat	*Triticum* *aestivum*	Misr 1	50%, 75%, and 100% FC	Corn stalk and rice husk	1%	Increased Chl-a and Ch- b, Car, RWC, grains per spike, 1000-grain weight, grain yield and harvest index	Hafez et al., 2021
		Glaxay 2013	30% WHC	Wheat straw	27.88 and 37.18 g kg^-1^	Increased plant height, number of fertile tillers, spike length, number of spikelets per spike, number of grains per spike and 1000-grain weight	Haider et al., 2020
		Galaxy-2013	–	Commercial charcoal	28 g kg^−1^ and 38 g kg^−1^	Plant height, spike length, number of spikelet’s per spike, number of grains per spike, 1000-grain weight, grain yield per plant, N, P and K contents in soil and microbial biomass	Zaheer et al., 2021
		Galaxy 2013	30% WHC	Wheat straw	3 and 5%	Increased plant height, fertile tiller count, spike length, grains per spike, 1000-grain weight, yield, water use efficiency, stomatal conductance, Chl-a, Chl-b, transpiration rate, photosynthetic rate, electrolyte leakage, H_2_O_2_, SOD, CAT and POD	Zulfiqar et al., 2022
Cabbage	*Brassica olerecae*	Yalova1	50% WHC	Commercial charcoal	5 and 10%	Increased FW and DW of shoot and root, Chl-a, Chl-b, total Chl, leaf RWC, CAT, POD and SOD activities and nutrient uptake Decreased H_2_O_2_, MDA and proline contents	Yildirim et al., 2021
Eggplant	*Solanum melongena*	Bonica F1	>30% WHC	Maize straw	6%	Increased plant height, aerial biomass, number of fruits per plant, flowering time and mean FW	Hannachi et al., 2023
Melon	*Cucumis melo*	–	60, 85, and 100% WHC	Palm leaves	0.24 and 0.36 kg m^-2^	Increased water use efficiency, FW and DW of root and shoot, root length, average fruit weight, fruit diameter, fruit flesh thickness, leaf N, Mn, K, Fe, Zn and Cu contents	Bagheri et al., 2019

RWC, relative water content; DW, dry weight; FW, fresh weight; Chl, chlorophyll; Car, carotenoid; ABA, abscisic acid; IAA,indole-3-acetic acid; GA, gibberellic acid; SOD, superoxide dismutase; CAT, catalase; POD, peroxidase; MDA, malondialdehyde; WHC, water holding capacity; FC, field capacity.

BC application enhanced the soil moisture holding capacity, net photosynthesis rate, water use efficiency and physiological, biomass and biochemical traits in milk thistle plants exposed to drought stress (Afshar et al., 2016). BC ameliorates drought-stressed soybean growth from the seedling stage to yield, including seed germination and biochemical and physiological traits (Hafeez et al., 2017; Zhang et al., 2020b; Gullap et al., 2024). The combined application of BC and silicon improved biomass- and yield-related traits in maize upon drought stress (Sattar et al., 2020). BC significantly enhanced physiological, biochemical and yield traits related to drought stress tolerance in maize (Hafez et al., 2020; Haider et al., 2020; Zaheer et al., 2021; Zulfiqar et al., 2022). It has been revealed that BC treatment strengthens the defense mechanisms of drought stressed plants. As with salt-stress responsive genes, many drought-tolerant and drought-susceptible genes have been reported in plants (Kaur and Asthir, 2017; Mahmood et al., 2019). The expression pattern and role of drought-tolerant and drought-susceptible genes have not been initiated under drought stress in plants with BC amendment. Hence, in-depth molecular experiments are urgently needed to underpin the expression pattern and role of drought-tolerant and susceptible genes in plants grown under BC.

### 8.3 Heavy metal stress

Contamination of agricultural soil by pollutants such as heavy metals has become a growing environmental problem worldwide that affects nutrient uptake, plant growth, and metabolism. Various measures have been used to remediate heavy metal contamination from soils, including the use of metal hyper accumulator plants, organic and inorganic amendments, and agricultural practices (Maharajan et al., 2022). Among these, organic amendments are effective techniques and eco-friendly methods to reduce plant uptake of high concentrations of heavy metals from heavy metal-contaminated soils. BC can absorb heavy metals from contaminated soil and reduce their toxic effects on plants. This has been demonstrated in many plant species (**Table 5**). The application of cotton stalk-derived BC increased growth, biomass, transpiration rate, sub-stomatal CO_2_ concentrations, photosynthetic rate, chlorophyll and carotenoid contents while reducing Cd concentrations and malondialdehyde content in shoot and root tissue under Cd toxicity (Younis et al., 2016). In a study by Woldetsadik et al. (2016), the application of poultry litter, cow manure, and coffee husk BC immobilized Cd in the soil and reduced the Cd concentration in plant tissues, while BC increased the growth and uptake of P in lettuce plants. In another study, BC deduced concentrations of Cd, Cu, Pb and increased the concentrations of P, Fe and Zn in shoot and root tissues of maize (Ahmad et al., 2018). Similarly, Miscanthus residues reduced nickel (Ni) contents in root, shoot and grains and increased the dry weight of shoot and root, photosynthetic rate, stomatal conductance, transpiration rate, yield, and several biochemical traits in maize upon Ni stress (Shahbaz et al., 2018).

**Table 5:** Effects of BC on plant growth and yield under heavy metal stress in various plants.

Name of the plant	Botanical name	Heavy metal level	BC sources	Level of BC	Positive and negative effects	References
Spinach	*Spinacia* *oleracea*	Cd (25, 50 and 100 mg kg^−1^)	Cotton stalks	3 and 5%	Increased growth, biomass, transpiration rate, substomatal CO_2_ concentrations, photosynthetic rate, Chl-a, Chl-b, total Chl, protein and Car contents Decreased Cd concentrations and MDA content in shoot and root tissue	Younis et al., 2016
		Mn (3.779 ppm), Ni (0.331 ppm), Zn (4.88 ppm), Cr (0.138 ppm) and Mg(111.7 ppm)	Cow manure and fresh sheep/goat manure	3, 5 and 10%	Increased the leaf area index, above-ground biomass, water use efficiency, root biomass Reduced Ni content in leaves	Tahir et al., 2018
		Pb (250 mg kg^−1^)	Waste material of vegetables and fruits	0.5%	Increased FW and DW weight of root and K contents Reduced Pb content in root	Zafar-ul-Hye et al., 2020
Lettuce	*Lactuca sativa*	Cd (50 mg kg^−1^)	Poultry litter, cow manure and coffee husk	7%	Increased plant growth, yield, and P uptake Reduced Cd concentration	Woldetsadik et al., 2016
Wheat	*Triticum* *aestivum*	Cd (2.86 mg kg^−1^), Zn (47.29 mg kg^−1^), Mn (68.31 mg kg^−1^) and Ni (5.33 mg kg^−1^)	Rice straw	1.5, 3.0 and 5.0%	Increased plant height, spike length, root, spike, grain and shoot biomass, grain yield, photosynthetic rate, Chl-a, Chl-b, transpiration rate, stomatal conductance, water use efficiency, Zn and Mn concentrations in shoots, roots, and grains and Si content, activities of SOD, CAT in shoot and root Decreased the Cd and Ni contents in shoot, root and grains and MDA content in shoot and root	Abbas et al., 2017
Tomato	*Solanum lycopersicum*	Cd (0.13 and 2 μg/ml)	Cotton stalk	1%	Increased soil pH, soil electronic conductivity, soil organic matter, DW of shoot and root, length of root and stem, Chl-a, Chl-b, total Chl, anthocyanin, Car and lycopene contents Reduced total Cd in soil, shoot Cd concentration	Abid et al., 2017
Maize	*Zea mays*	Cu (3474 mg kg^−1^) Fe (25 962 mg kg^−1^) Mn (1413 mg kg^−1^) Pb (1360 mg kg^−1^) Zn (11 239 mg kg^−1^) Cd (27.8 mg kg^−1^)	Date palm tree waste	1, 2 and 3%	Increased P, Fe and Zn concentration in shoot and root Reduced Cd, Cu, Pb concentration in shoot and root tissue	Ahmad et al., 2018
		Ni (77 mg kg^−1^)	Miscanthus plant residues	2%	Increased DW of shoot and root, grain yield, photosynthetic rate, stomatal conductance, transpiration rate, CAT, APX and DHAR activities, ABA in leaves, protein, fiber, fat, starch Reduced MDA and H_2_O_2_ activities in leaf and polyphenol, Ni contents in root, shoot and grains	Shahbaz et al., 2018
Chinese cabbage	*Brassica chinensis*	Cd (41 mg kg^−1^)	Rice straw, rice hull and maize stover	1.5 and 3%	Increased FW and DW of shoot and root, length of shoot, root and leaves Reduced Cd content in shoot and root tissue	Bashir et al., 2018
		Cd (20 mg kg^−1^)	Wheat straw	5%	Increased plant biomass Reduced Cd contents in fruits	Sun et al., 2020
Chinese flowering cabbage	*Brassica parachinensis*	Cd (1.19 mg kg^−1^)	*Pennisetum hydridum* straws	2%	Increased vegetable growth, plant height, root length, root FW, above-ground FW and reduced Cd content in the roots and above ground tissues	Li et al., 2021b
Sunflower	*Helianthus annuus*	Ni (77 mg kg^−1^)	Miscanthus residues	2%	Increased DW of shoot and root, grain yield, photosynthetic rate, stomatal conductance, transpiration rate, CAT, APX and DHAR activities, ascorbic acid in leaves, protein, fiber, fat, starch Reduced MDA and hydrogen peroxide activities in leaf and polyphenol, Ni contents in root, shoot and grains	Shahbaz et al., 2018
Pea	*Pisum* *sativum*	Pb (1000 mg kg^−1^)	–	2%	Increased DW of shoot and root, grain yield, plant height, RWC, Chl-a, Chl-b, protein, fat, fiber, carbohydrate, Fe, Zn and Mn contents, APX, SOD, CAT and DHAR activities Reduced Pb content in root and shoot and polyphenols, MDA, H_2_O_2_ and O_2_	Haider et al., 2019
Peanuts	*Arachis hypogaea*	Cd (1 mg kg^−1^)	Peanut vine and rice straw	5%	Increased soil pH, Chl content, soluble sugars, proline, soluble protein and crude fat Reduced Cd content in the root, above-ground tissues, shell and seed	Chen et al., 2020
Rapeseed	*Brassica napus*	Cd (0.28 mg kg^−1^), Cr (11.30 mg kg^−1^), Cu (3.60 mg kg^−1^), Zn (17.94 mg kg^−1^), Ni (1.38 mg kg^−1^), Pb (5.84 mg kg^−1^), Co (0.10 mg kg^−1^) and Fe (136.38 mg kg^−1^)	*Acacia nilotica* woodchip	1 and 2%	Increased FW of shoot and root, Chl-a, Chl-b, total Chl, total pigments, Car, lycopene concentration, APX, POD and CAT activities. Reduced Cd, Pb, Ni and Cu in soil and shoot and roots	Kamran et al., 2020
Barley	*Hordeum vulgare*	Cd (1.25-1.91 mg kg^−1^), Cu (200-451 mg kg^−1^), Pb (118-211 mg kg^−1^) and Zn (67.2-134 mg kg^−1^)	Miscanthus residues	2%	Increased shoot and root biomass and reduced Cu, Pb, Cd and Zn contents in shoot and root tissues	Medynska et al., 2020
Green pepper	*Capsicum annuum*	Cd (20 mg kg^−1^)	Wheat straw	5%	Increased plant biomass and reduced Cd contents in fruits	Sun et al., 2020
Eggplant	*Solanum melongena*	Cd (20 mg kg^−1^)	Wheat straw	5%	Increased plant biomass and reduced Cd contents in fruits	Sun et al., 2020

DW, dry weight; FW, fresh weight; Chl, chlorophyll; Car, carotenoid; ABA, abscisic acid; APX, ascorbate peroxidase; DHAR, dehydrogenase reductase; SOD, superoxide dismutase; CAT, catalase; POD, peroxidase; MDA, malondialdehyde.

BC derived from wheat straw increased plant biomass and reduced Cd contents in fruits of green pepper and eggplant (Sun et al., 2020). Overall, BC could be effective in immobilizing heavy metals in the soil and reducing heavy metal uptake and accumulation in plant tissues. In general, HM uptake by roots is facilitated by various metal transporters (Maharajan et al., 2022). However, the role of heavy metal transporters has not yet been reported in BC-treated plant tissues. Hence, environmental researchers should collaborate with plant molecular biologists to initiate in-depth molecular experiments in this field, which may help to understand the accurate role of BC in plant growth and yield. Apart from this, BC derived from heavy metal accumulator plant residues has not yet been used for any experiments. Hence, researchers can try to derive BC from heavy metal accumulator plant tissues and apply them to identify the effect of BC on plant growth and yield.

## 9 Conclusion and future perspectives

The utilization of BC, derived from agricultural and forestry residues, aligns with global sustainability goals by converting waste materials into valuable resources that enhance agricultural output. Synthesis techniques, BC processing, soil amendments, and applications to alleviate abiotic stressors in plants have received a lack of research attention. Hence, this review compiles the conversion of residual biomass into valuable BC amendments for soil and crop improvement upon abiotic stressors. Due to the post pandemic financial crisis, low-cost soil amendments are mandatory to increase crop production to overcome worldwide food scarcity. BC application is a promising strategy to promote soil–plant enrichment for the production of highly nutritious crops and yields, ameliorate plant abiotic stresses, controlled usage of hazardous synthetic fertilizer-based soil amendments to enhance sustainable agriculture. BC has a positive impact on soil structure, quality, and physico-chemical properties such as BD, pH, CEC, porosity, nutrient balance, WHC, and aeration. The optimal ratio of BC formulations has been potentially enriching yields under plant- and soil-specific constraints, limited water, nutrients and adverse conditions. Also, offering a promising avenue for enhancing global food security and environmental health. The recommended dosage of BC for quality improvement under specific soil and plant species is not yet well defined. Moreover, research must be focused on BC at low doses, and high efficacy is crucial to maximize farmer-friendly, cost-effective BC applications for several cropping systems.

The synergistic effects of BC along with compost, fertilizers and beneficial soil microbes that stimulate crop growth and soil fertility remain unclear. Although, long-term BC risk management and life cycle assessments are not yet completely clarified. The discrepancy between field and laboratory experiments regarding physico-chemical properties, soil quality, abiotic stress, environmental impacts, and plant growth efficiency should be scrutinized. BC production parameter optimization, functionalization, elucidation of BC augmenting mechanisms, integration of multi-omics technologies, data-driven and machine learning methods would contribute to BC applications for soil fertility and cost-effective high-yielding production of valuable crops in sustainable agricultural management. While initial production and application costs can be high, the long-term savings, enhanced crop resilience, and potential carbon credits make BC a viable option. However, further technological advancements and policy support are essential to encourage broader adoption. As per our knowledge, physiological and biochemical modifications by BC under abiotic stress have been identified, while molecular identification and characterization in this field remain underexplored. These areas of research should be prioritized by plant and environmental scientists, as they could provide deeper insights into plant development, food security, and sustainable agricultural practices. In summary, increasing soil health along with sustainable crop productivity and profitability under challenging environmental conditions can be significantly influenced by BC soil amendment technologies. Moreover, additional investigation is required to implement the machine learning approach on BC-based soil amendment, the alleviation of abiotic stress for sustainable crop production, soil fertility management, and hence light up BC industrialization.
